# Resveratrol as sensitizer in colorectal cancer plasticity

**DOI:** 10.1007/s10555-023-10126-x

**Published:** 2023-07-29

**Authors:** Aranka Brockmueller, Anjana Sajeev, Lenka Koklesova, Samson Mathews Samuel, Peter Kubatka, Dietrich Büsselberg, Ajaikumar B. Kunnumakkara, Mehdi Shakibaei

**Affiliations:** 1 Chair of Vegetative Anatomy, Institute of Anatomy, Faculty of Medicine, LMU Munich, Pettenkoferstr. 11, D-80336 Munich, Germany; 2 Cancer Biology Laboratory, Department of Biosciences and Bioengineering, Indian Institute of Technology (IIT) Guwahati, Guwahati, Assam 781039 India; 3 Clinic of Gynecology and Obstetrics, Jessenius Faculty of Medicine, Comenius University in Bratislava, Kollarova 2, 03601 Martin, Slovakia; 4 Department of Physiology and Biophysics, Weill Cornell Medicine-Qatar (Medbay), Education City, Qatar Foundation, 24144 Doha, Qatar; 5 Department of Medical Biology, Jessenius Faculty of Medicine, Comenius University in Bratislava, Mala Hora 4, 03601 Martin, Slovakia

**Keywords:** Colorectal cancer, Chemoresistance, Chemosensitization, Multidrug resistance, Resveratrol, Cancer cell plasticity

## Abstract

**Abstract:**

Despite tremendous medical treatment successes, colorectal cancer (CRC) remains a leading cause of cancer deaths worldwide. Chemotherapy as monotherapy can lead to significant side effects and chemoresistance that can be linked to several resistance-activating biological processes, including an increase in inflammation, cellular plasticity, multidrug resistance (MDR), inhibition of the sentinel gene p53, and apoptosis. As a consequence, tumor cells can escape the effectiveness of chemotherapeutic agents. This underscores the need for cross-target therapeutic approaches that are not only pharmacologically safe but also modulate multiple potent signaling pathways and sensitize cancer cells to overcome resistance to standard drugs. In recent years, scientists have been searching for natural compounds that can be used as chemosensitizers in addition to conventional medications for the synergistic treatment of CRC. Resveratrol, a natural polyphenolic phytoalexin found in various fruits and vegetables such as peanuts, berries, and red grapes, is one of the most effective natural chemopreventive agents. Abundant *in vitro* and *in vivo* studies have shown that resveratrol, in interaction with standard drugs, is an effective chemosensitizer for CRC cells to chemotherapeutic agents and thus prevents drug resistance by modulating multiple pathways, including transcription factors, epithelial-to-mesenchymal transition-plasticity, proliferation, metastasis, angiogenesis, cell cycle, and apoptosis. The ability of resveratrol to modify multiple subcellular pathways that may suppress cancer cell plasticity and reversal of chemoresistance are critical parameters for understanding its anti-cancer effects. In this review, we focus on the chemosensitizing properties of resveratrol in CRC and, thus, its potential importance as an additive to ongoing treatments.

**Graphical abstract:**

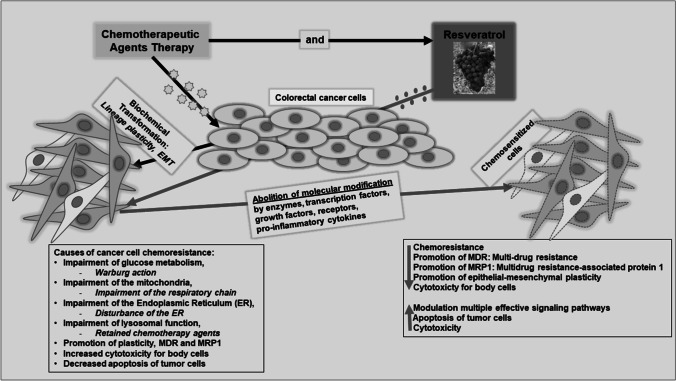

## Introduction

Across populations, physicians are faced with treating colorectal cancer (CRC), ranked third worldwide for cancer incidence and cancer-associated deaths [1], according to a summation of case numbers from 185 countries. Worldwide, more than 1.18 million people were affected by CRC in 2020 [1], and in 2022, there were over 151,000 new diagnoses and approximately 53,000 CRC-related deaths in the USA alone [2]. These collected data include neoplasms of both the colon and rectum. In the search for causes, the age-associated adenoma-carcinoma theory [3], in which malignant degenerations arise from benign precursors during life, is very relevant. Moreover, as a multifactorial process, CRC is often only detected at an advanced stage of the disease, as there are no clear and conspicuous signs. If the cells have already metastasized, the treatment process is highly time-consuming and rarely leads to the expected therapeutic success [2]. Therefore, early detection of a possible CRC in its benign or early-stage CRC should be encouraged, paving the way for appropriate and effective intervention.

At the molecular level, CRC development and spread are initiated and accelerated primarily by pro-inflammatory processes [4]. After developing a primary tumor, cancer cells can also detach from it and spread via lymphogenic or hematogenic routes to other organs. In the case of CRC, this leads primarily to portal vein-type metastases, 30–60% of which often [5] manifest themselves first in the liver. Unimpeded metastatic growth leads to organ failure and is one of the frequent causes of death in cancer patients. To prevent or treat metastasis, CRC patients receive chemotherapy in most cases after colorectal surgery. Due to the aggressiveness of the disease, this usually consists of several components, such as folinic acid and 5-fluorouracil (5-FU) combined with oxaliplatin (FOLFOX) or irinotecan (FOLFIRI). Tumor cells are becoming increasingly chemoresistant to common chemotherapeutic agents used against various cancers, with a high frequency of recurrence due to the modification of multiple metabolic pathways [6]. In this regard, increasing B-cell lymphoma 2 (Bcl-2) expression and thus inhibition of apoptosis, enhancing expression of hypoxia-inducible factor (HIF)-1 and thus tumor cell survival, raising the expression of multidrug resistance (MDR) protein 1, epithelial-mesenchymal-plasticity, sensitizes the cell to trans-differentiation, and hence, the promotion of drug efflux and initiation of medication inhibition are of central importance. Increasing the expression of the pro-inflammatory transcription factor ‘nuclear factor kappa-light-chain-enhancer of activated B-cells’ (NF-κB), which regulates the expression of inflammatory and cytoprotective genes, inhibiting the cellular tumor antigen p53 (p53) and thus inducing cell survival, which is a significant obstacle to the treatment of cancer patients [7–10] are further key examples. Moreover, undesirable side effects, such as the development of acquired resistance of CRC cells to chemotherapeutic agents, lead to a significant decrease in the efficacy of cytostatic drugs; thus, cancer continues to epithelial-mesenchymal-plasticity and spread despite treatment [6, 11]. Indeed, classical drugs with limited efficacy have not been able to solve this dramatic and widespread problem, so scientists are constantly searching for agents without side effects and for innovative solutions to improve cancer treatment.

In addition, the ever-increasing importance of people’s lifestyle habits, especially among modern populations, is now recognized as the most critical cause of CRC, particularly their diet. In this context, regular consumption of fermented foods, cigarette smoking, processed meats, alcohol, increased body mass index, and lack of exercise are considered unhealthy and have a favorable effect on the development of CRC [12]. Indeed, it was shown that a biologically balanced diet, exceptionally high in plant foods and fruits, can significantly reduce the risk of cancer [13, 14]. Therefore, interest is increasingly directed toward natural substances, which have been used for many decades in medical therapy to prevent various diseases, including cancer [15–17].

Whether secondary plant compounds can be used in the management of human diseases has been investigated for many years. These natural substances possess a poly-target action capability and thus have more versatile attack options in parallel than synthetically developed one’s mono-target drugs [18–20]. Against this backdrop, resveratrol is a well-researched plant-derived polyphenol, preventing the onset and advancement of CRC. This phytopharmaceutical occurs naturally in berries, grapes, and nuts [21–23], protecting the fruit from fungal infestation, oxidative processes, aging, and spoilage. It has already shown numerous relevant medical effects in mammalian and human cells. For example, in the field of cardiovascular diseases, a vasodilatory effect on a blood vessel by resveratrol-induced nitric oxide (NO)-mediated mechanisms is known [24, 25] and has the effect as a phytoestrogen [26–30]. In addition, resveratrol has protective and regeneration-promoting effects on nerves after injuries [31] and suppresses inflammatory cytokine storms related to chronic obstructive pulmonary disease [32]. Due to its high overall prevalence, resveratrol is a topic of current cancer research. Its effects on cancer cells and patients are being studied *in vitro*, *in vivo,* and clinically [33–35]. Specifically, this phytopharmaceutical has shown significant immunomodulatory potential [36–39] and immune system-balancing effects concerning tumor necrosis factors (TNFs) and interleukins (ILs) in both healthy and lymphoma patients [40]. Moreover, Resveratrol is a poly-target agent capable of modifying several cell signaling cascades, selectively exerting cytotoxicity on cancer cells and able to attenuate cell metastasis by suppressing the epithelial-to-mesenchymal transition (EMT) plasticity signaling [41] and, simultaneously, no toxicity on normal cells [42].

In this review, we address the anti-CRC and chemosensitization mechanisms of resveratrol, focusing on tumor cell plasticity (phenotypic trans-differentiation of cancer cells), which plays an essential role in transformation, progression, malignancy, metastasis, and also therapy resistance of cancer cells to conventional drugs. Consequently, the prevention of this dynamic developmental process is a key prerequisite for the prevention and improvement of clinical treatment success in cancer patients.

## Goal of the review

This review aims to provide an overview of the high prevalence of CRC, focusing on the importance and fundamental role of cellular plasticity in frequent metastasis and the consequences of the development of resistance in CRC cells to classical chemotherapeutic agents. In this context, the anti-CRC potential of resveratrol, based on the suppression of EMT-plasticity, is reviewed, providing a pathway for overcoming chemoresistance that may represent a groundbreaking new advance in the treatment of CRC.

## Resveratrol, a plant-derived polyphenol

**Fig. 1:**
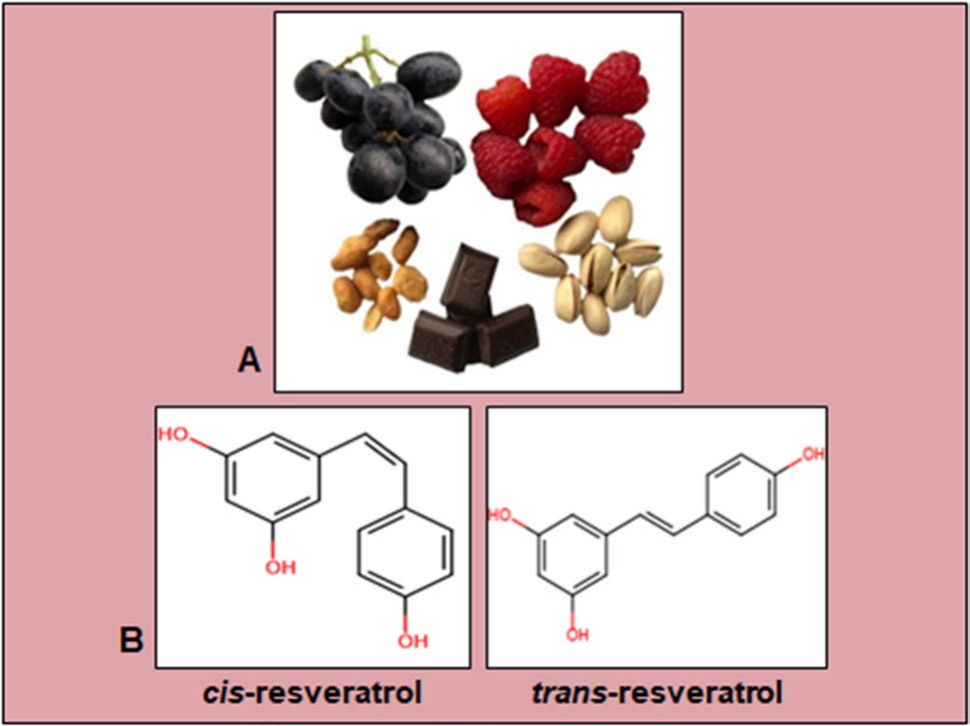
Description of resveratrol. **A** Exemplary photo of resveratrol-containing food products. **B** Chemical structure of *cis*-resveratrol and *trans*-resveratrol, composed with a creator from Fisher Scientific (Schwerte, Germany)

Resveratrol is a plant stilbene with two phenolic rings linked by a double styrene chain, and it exists in two isoforms, *cis* and *trans* (Fig. 1). The *trans*-isoform is the most abundant and best studied and is catalyzed by the enzyme stilbene synthase [43]. Resveratrol was initially detected in the roots of white hellebore (*Veratrum grandiflorum*) and is now found in over 70 widely distributed plant species, including red wine grapes, cranberries, peanuts, and root extracts of the weed *Polygonum cuspidatum* [44, 45].

### Resveratrol‘s natural sources and chemical properties

The phytoalexin resveratrol was extracted for the first time from the herbaceous plant *Veratrum grandiflorum* by the Japanese scientist Takaoka in 1939 [46]. It is a solid, alcohol-soluble ingredient alternatively known as 3,5,4′-trihydroxystilbene [47], with the sum formula being C_14_H_12_O_3_ [48]. Structurally, both *cis*- and *trans*-isomer exist, with the trans-form (Fig. 1) being more abundant and convertible to the *cis*-form [49, 50]. Numerous different targets of resveratrol have been identified, including integrin-receptors, estrogen-receptors, and sirtuins (SIRT) [51–53].

The best-known source of naturally occurring resveratrol is grapes of any color (Fig. 1), and the ingredient has been detected in bilberries, cranberries, and strawberries [54]. Consequently, this secondary phytochemical is also found in juices produced from these fruits and grape-based alcoholic beverages such as wine and champagne [54]. Furthermore, nuts such as peanuts and pistachios contain resveratrol, as does dark chocolate (Fig. 1) [55], which the presence of this plant component in cocoa beans could explain. In addition, some plants like *Polygonum cuspidatum* are particularly resveratrol-containing, leading to the occurrence of the Itadori tea brewed from it in Asia [56].

The legitimate inferential question is: Why has resveratrol been detected in many natural products? The answer is astonishing: The production of resveratrol is stimulated in plants whenever they are exposed to the stress of different genesis, for example, UV-radiation [22], ozone-exposition, or pathogenic confrontation [57]. Overall, this plant-native polyphenol balances the consequences of harmful environmental influences, protects the vegetables from fungal attack, parasite infestation, over-ripening, and rot [56], and thus represents a natural survival strategy. As humans are also permanently exposed to environmental influences of various kinds and often live a lifestyle that is detrimental to their health, research is constantly being conducted to determine whether and how to use resveratrol’s protective properties for the benefit of humankind.

### Bioavailability, absorption, and metabolism of resveratrol

According to previous publications, the absorption of resveratrol by the oral route in man is around 75-80% and presumed to be predominantly by transepithelial diffusion *in vitro* and *in vivo* [58, 59]. It has already been reported that resveratrol is distributed in high concentrations in tissues. Indeed, resveratrol accumulated nearly 40-fold in the human CRC cell line Caco-2 *in vitro* versus medium [60], concluding that intestinal cells are a primary target for this polyphenol. Because of the strong metabolism in the digestive tract and hepatobiliary system, with a short half-life of about 1.5 hours [60], bioavailability by oral ingestion is relatively limited, less than 1%, which does not change with increasing intakes. Biochemical studies have shown that resveratrol’s major degradation products in blood plasma and urine are glucuronides, di-hydro-resveratrol conjugates, as well as sulfates [61, 62] and about 50–60% of the ingested phytopharmaceutical is eliminated from the body in urine [58]. Encouragingly, the ingestion of 500mg resveratrol in tablet form, resulting in plasma concentrations of about 70ng/ml, was reported to be well-tolerated and safe [63]. However, some adverse effects, such as diarrhea, nausea, anemia, vomiting, and flatulence, were reported when resveratrol was administered in high doses [64, 65].

Optimizing this topic, a higher bioavailability of this compound has been achieved by creating more sophisticated preparations, including nanoparticles and nano-constructed lipid vehicles incorporating resveratrol, including verification of the efficacy of the association in recovering chemosensitivity. In addition, the oral bioavailability of resveratrol incorporated in casein nanoparticles is tenfold increased compared to the administration of the polyphenol in the form of an oral suspension [66, 67]. Furthermore, enhancement of resveratrol’s bioavailability in rats has already been targeted by treatment with 3,5,4’-tri-O-acetyl-resveratrol, an acetylated resveratrol precursor that can be hydrolyzed in cells to free *trans*-resveratrol [68, 69]. Research in this area is still in the preliminary stages, but it is anticipated and promising that these novel and innovative nano-transporters will yield significant benefits.

Moreover, in addition to organs such as the intestine and the hepatic system, the vital colon microflora is suspected of playing a central role in resveratrol metabolism [61]. Considering the significant and indispensable importance of the intestinal microbiota for many essential body functions, and here for the metabolism of resveratrol, it could be of fundamental and crucial relevance, as it has already been demonstrated for other natural products such as soy isoflavone and lignans [70, 71]. Altogether, the high accumulation and concentration of resveratrol in the enterocytes of the colon epithelium suggest that this area of the colon [72], because of the intestinal microflora, probably plays an essential role in the bioactivity of resveratrol metabolism.

## Resveratrol’s versatile anti-carcinogenic effects

### Resveratrol’s effect on different cancer types

Research into resveratrol’s effects and detailed mechanisms of action on different cancer cell types and lines has been of high scientific interest for decades. To illustrate this with some examples, a treatment with this natural polyphenol disrupts the cell cycle flow of cervical cancer cells [73], reduces the inflammation-related invasiveness of gastric cancer cells [41, 74], and initiates apoptosis in prostate cancer cells [75–77]. Moreover, as a multitargeting agent in CRC cells, this active plant compound prevents invasion, proliferation, and metastasis [52]. It averts EMT-associated plasticity, necessary for metastasis, with a parallel promotion of apoptosis and being a chemosensitizer for treating cancers [78–81]. Resveratrol modulates epigenetic changes in tumor cells, as it can stimulate the ATP2A3 gene, leading to down-regulation of histone deacetylase (HDAC) and thus HDAC2 expression in the nucleus, or through deoxyribonucleic acid (DNA) methylation, histone modification, non-coding messenger ribonucleic acid (mRNA), and telomerase levels, leading to suppression of cancer spread [81, 82]. In line with this, a clinical study showed that a daily oral intake of 0.5–1g resveratrol could have anti-carcinogenic effects in the human gastrointestinal tract and, at the same time, be well-tolerated by cancer patients [35].

Resveratrol has also been shown to inhibit cellular processes associated with the development of tumors through mutation. Indeed, it has been reported that resveratrol possesses several active anti-oxidant capacities [83], acts as an active scavenger of hydroxylenes/superoxides [84] and significantly blocks the formation of free radicals caused, for example, by 12-O-tetradecanoylphorbol-13-acetate (TPA) in cancer cells [85]. Furthermore, resveratrol also contributes to protection against lipid peroxidation in cell membranes and DNA damage caused by the release of reactive oxygen species (ROS) [86, 87]. In addition, the phytopharmaceutical has been found to have mutation-inhibiting and anti-carcinogenic effects, such as preventing the mutagenicity of N-methyl-N’-nitro-N-nitrosoguanidine in *Salmonella typhimurium* [88].

**Fig. 2:**
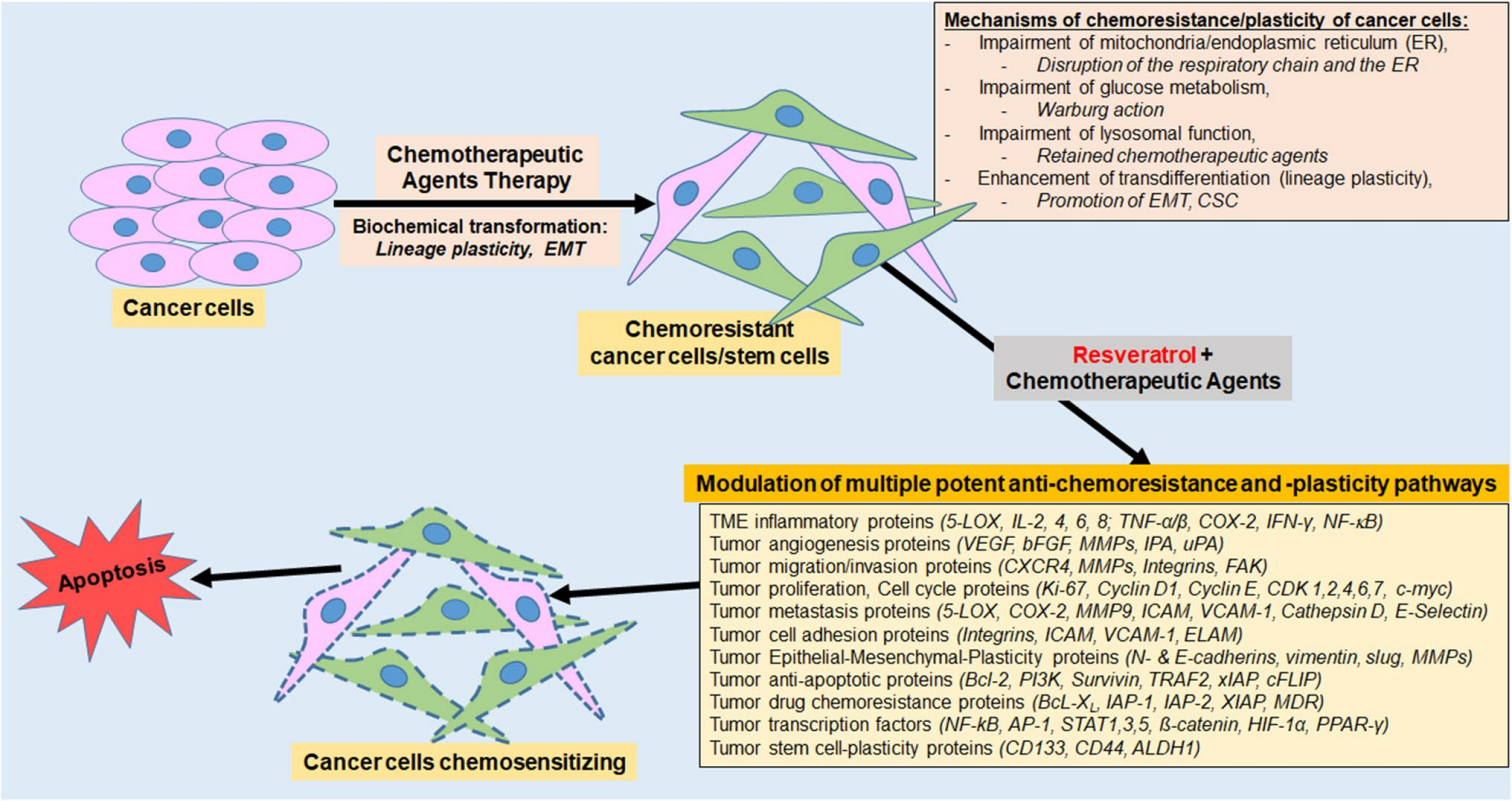
Generation of resistance in cancer cells and its recovery by co-treatment with resveratrol. Cancer cells become resistant to several chemotherapeutic medications because of modifications in different regulatory pathways. Co-treatment with resveratrol and ongoing chemotherapeutic agents transforms these biological changes by simultaneously affecting multiple signaling pathways, resulting in the chemosensitization of tumor cells to chemotherapy agents. Abbreviations: 5-LOX, 5-lipoxygenase; ALDH, aldehyde dehydrogenase; AP-1, activator protein 1; Bcl-2, B-cell lymphoma 2; Bcl-xL, B-cell lymphoma extra-large; bFGF, basic fibroblast growth factor; CSC, cancer stem cell; CD, cluster of differentiation; CDK, cyclin-dependent kinase; cFLIP, cellular FLICE-inhibitory protein; cMyc, Cellular myelocytomatosis oncogene; COX, cyclooxygenase; CXCR, C-X-C chemokine receptor; ELAM, Endothelial Leukocyte Adhesion Molecule; EMT, epithelial-to-mesenchymal transition; ER, endoplasmic reticulum; FAK, focal adhesion kinase; HIF, hypoxia-inducible factor; IAP, inhibitor of apoptosis protein; ICAM, intercellular adhesion molecule; IFN, interferone; IL, interleukin; IPA, indolephenoxyacetamide; Ki-67, Kiel-antigen 67; MDR, multidrug resistance; MMP, matrix metalloproteinase; NF-κB, nuclear factor kappa-light-chain-enhancer of activated B-cells; PI-3K, phosphoinositide 3-kinase; PPAR-γ, Peroxisome proliferator-activated receptor gamma*;* Slug, SNAI2; snail homolog 2; STAT, signal transducer and activator of transcription; TME, tumor microenvironment; TNF, tumor necrosis factor; TRAF, TNF receptor-associated factor; uPA, urokinase-type plasminogen activator; VCAM, vascular cell adhesion molecule; VEGF, vascular endothelial growth factor

Cancer cells can become chemoresistant to various chemotherapeutic agents due to modifications in diverse biological processes in the subcellular signaling pathways [7, 8]. Resveratrol has great promise for targeting several molecular and cell signaling pathways. It has already been explored in various preclinical and clinical approaches as a chemosensitizer drug for combined therapy with standard drugs for diverse types of cancer [89, 90]. Figure 2 provides an overview of the beneficial properties of the combination treatment of standard chemotherapeutic substances with resveratrol and its various potential biological pathways involved in the chemosensitization of tumor cells. Table 1 lists recent research findings on the different subcellular signaling pathways and their mechanisms of resveratrol having anti-inflammatory, anti-proliferative, anti-metastatic, anti-oxidative, immunomodulatory, and pro- or anti-apoptotic effects in various *in vitro* or *in vivo* studies in cancer and healthy cells.

**Table 1:** Various subcellular signal-modulating networks in cancer and healthy cells as resveratrol targets

Subcellular signaling / Mechanism		References
*Down-regulation of*	*Transcription factor signaling pathway*	
	- NF-κB signaling pathway	[91–102]
	- HIF-1α signaling pathway	[93, 103–113]
	- MAPK signaling pathway	[114–119]
	- AP-1 signaling pathway	[120, 121]
	- STAT3 signaling pathway	[122–124]
	- β-Catenin signaling pathway	[125–127]
	- Cell cycle signaling pathway	[128, 129]
	- Growth factor signaling pathway	[92, 130, 131]
	- Mitochondrial signaling pathway	[132, 133]
	- Inflammation signaling pathway	[134, 135]
	- Oxidative signaling pathway	[136, 137]
	- Mutagenesis signaling pathway	[138, 139]
	- Angiogenesis signaling pathway	[140]
	- Plasticity/Migration signaling pathway	[141–145]
	- Estrogen signaling pathway	[146, 147]
	- RANKL signaling pathway	[148–152]
	- Apoptosis signaling pathway	[153–159]
*Up-regulation of*	*Transcription factors signaling pathway*	
	- Sox9 signaling pathway	[154, 155, 160–162]
	- Scleraxis signaling pathway	[163]
	- PPAR-γ/RUNX2 signaling pathway	[164–170]
	- PI3K/Akt/mTOR signaling pathway	[171–173]
	- p53 signaling pathway	[174, 175]
	- Autophagy signaling pathway	[176, 177]
	- Apoptosis signaling pathway	[178, 179]
	- Estrogen signaling pathway	[26–30]
	- Maintenance of the cellular signaling pathway	[180–184]
	- Immunomodulatory signaling pathway	[36–39]

Abbreviations: *Akt* protein kinase B, *AP-1* activator protein 1, *HIF* hypoxia-inducible factor, *mTOR* mammalian target of Rapamycin, *NF-κB* nuclear factor kappa-light-chain-enhancer of activated B-cells, *PI-3K* phosphoinositide 3-kinase, *PPAR-γ Peroxisome proliferator-activated receptor gamma*, *RANKL* receptor activator of NF-kappaB ligand, RUNX2 Runt-related transcription factor 2, *SOX9* SRY-Box Transcription Factor 9, S*TAT3* signal transducer and activator of transcription 3, *MAPK* mitogen-activated protein kinase

### Resveratrol modulates inflammation and acts anti-carcinogenic in CRC cells

Acute inflammation is activated by immune-specific cells, especially in infections and allergies. Thus, it is part of the healthy immune system in the body, and it lasts only for a short time. However, if the inflammation lasts longer, it becomes chronic [185]. Key mechanisms such as chronic inflammation and the associated induction of angiogenesis, metabolizing enzymes, oxidation, cell cycling, cell plasticity, and anti-apoptotic proteins are among the most important prerequisites for the development of chronic diseases, including colitis and CRC, making agents to prevent and inhibit inflammation in the tissues, like colon and thus prevent colitis and CRC of particular interest. Well-documented inflammatory signaling pathways associated with the pathogenesis of colitis-related CRC include NF-κB, IL-6/STAT3, cyclooxygenase (COX)-2/prostaglandin E_2_ (PGE_2_), and IL-23/*T helper 17* cells (Th17) [186]. More specifically, pro-inflammatory chemokines and cytokines, such as IL-1, -6, -8, TNF-α, and TNF-β, are produced very rapidly by injured body tissues and can trigger a variety of inflammatory responses and the expression of pro-inflammatory transcription factors, such as NF-κB, mitogen-activated protein kinase (MAPK), signal transducer and activator of transcription (STAT) 3, HIF-1α, activator protein-1 (AP-1), and nuclear factor erythroid 2–related factor 2 (Nrf2), and their secondary inflammatory substances such as inflammatory mediators, such as matrix metalloproteinases (MMPs), 5*-*lipoxygenase (5-LOX), COX-2, and also the production of ROS [187–191]. Inflammatory bowel disease (IBD) and Lynche’s syndrome have been shown to contribute to significantly increased development and pathogenesis of CRC, suggesting an intense interaction between inflammation and cancer development. In addition, experimental animal models of IBD have clearly demonstrated that resveratrol is a beneficial agent for the management of IBD [192].

Furthermore, one of the most critical pro-inflammatory transcription factors in inflammatory tissues is NF-κB, expressed by multiple cancers such as CRC [193]. This transcription factor induces genes involved in cell survival, cell adhesion, inflammation, differentiation, and growth. NF-κB is activated by various influences such as carcinogens, phorbol ester, pro-inflammatory agents, cigarette smoke, and cytokines such as IL-1β and TNF-α or TNF-β [194]. These factors promote the dissociation of NF-κB inhibitor alpha (IκBα) through phosphorylation, and the activated NF-κB migrates from the cytoplasm to the nucleus, leading to the binding and activation of transcription of target genes essential for the development of aggressive cancers. The expression of proliferation proteins (cyclin D1, Kiel-antigen 67 (Ki-67)), apoptosis suppressor proteins (Bcl-2 and B-cell lymphoma extra-large (Bcl-xL)), and proteins responsible for metastasis (MMPs, COX-2, CXCR4) as well as angiogenesis (vascular endothelial growth factor (VEGF)) are promoted by NF-κB [194]. Similarly, IL-6/JAK/STAT3 signaling pathway potently activates inflammatory response via tumor-infiltrating immune cells in the tumor immune microenvironment in CRC. Moreover, IL-6/JAK/STAT3 signaling up-regulates downstream target genes with anti-apoptotic and proliferative effects, promotes plasticity, invasion, and metastasis of cancer cells and angiogenesis, and induces cancer resistance [195, 196]. The COX-2/PGE_2_ signaling has been closely associated with all stages of colorectal carcinogenesis. The role of COX-2 and its product PGE_2_ in the pathogenesis of CRC is based on the function of fibroblasts from the mesenchymal (stromal) layer, which are the main target of cytokines e.g. TNF-α and IL-1β. Fibroblasts from non-neoplastic colorectal tissue are an important source of COX-2 expression that is well-validated as one of the most important risk factors of CRC [197]. Finally, IL-23 receptors play a crucial role in chronic inflammatory diseases due to their function in the processes of differentiation of Th17. IL-23 up-regulates PGE_2_ levels and Th17 cell function that include expression increase of inflammatory cytokines, such as IL-17A, IL-17F, IL-21, and IL-22. For these reasons, the IL-23/Th17 signaling is strongly included in the pathogenesis of colitis-associated CRC [198].

Chemopreventive phytochemicals such as resveratrol inhibit several pro-inflammatory-related activations of transcription factors, cytokines, chemokines, proteins, and enzymes [199–205]. Much evidence suggests that resveratrol is a multi-factorial bioactive phytochemical with numerous beneficial preventive effects on subcellular biological pathways, especially anti-inflammatory effects by inhibiting pro-inflammatory cytokines (IL-1β, TNF-α, and TNF-β), the pro-inflammatory transcription factor NF-κB and thus NF-κB-promoted end-proteins. A recent molecular docking study documented that resveratrol could be effective against CRC by targeting NF-κB signaling [206], and in this regard, our group described resveratrol’s NF-κB suppression by resveratrol associated with an anti-inflammatory mode of action in CRC [207]. Furthermore, a seven-day treatment with resveratrol (10mg/kg body weight) suppressed the dextran sulfate sodium-induced inflammatory colon injury via down-regulation of NF-κB, STAT3, ERK, and iNOS expressions in IRC mice [208]. Combinational application of resveratrol with 5-FU inhibited Akt/STAT3 signaling, which was associated with pro-apoptotic effects and increased anti-telomerase activity in human CRC cells [209]. Using DLD1 and HCT15 CRC cells, resveratrol inhibited cancer growth by targeting the Akt/STAT3 signaling pathway. These anti-cancer effects of resveratrol correlated with pro-apoptotic effects and blockage of the G1 phase cell cycle in cancer cells [123]. In addition, the resveratrol treatment sensitized HT-29 and SW620 CRC cell lines to 5-FU (via increased oxidative stress) through the down-regulation of Akt and STAT3 signal proteins [210]. Another study showed the inhibitory effect of resveratrol on the COX-2/PGE_2_ signaling pathway (decreased both miRNA and protein levels) and consequent anti-cancer efficacy in HCT-116 human CRC cell lines [211]. A similar result was found in a Serra et al. study describing the inhibition of COX-2/PGE_2_ signaling by resveratrol (also isoenzymes of NO synthase (iNOS) expression) in HT-29 colon epithelial cells [212]. The combination of resveratrol and ginkgetin synergistically attenuated the 5-FU-induced inflammation in HT-29 colon cancer xenograft nude mice through decreased expressions of COX-2 and inflammatory cytokines [213]. A micro-immunotherapy sequential medicine including resveratrol showed significant immunomodulatory effects on human macrophages via several cytokine-induced signaling pathways (including IL-23) and consequent tumor-suppressive efficacy using *in vitro* 2D and 3D spheroid models and animal xenograft colon carcinoma experimental approach [214]. Furthermore, the phytopharmaceutical possesses potential anti-cancer functions, such as CRC cell survival reduction, activation of apoptosis (caspase-3), inhibition of invasion, and preventing of EMT-plasticity in the CRC tumor microenvironment (TME) *in vitro* and *in vivo* (Table 2) [215–234].

**Table 2:** The modulatory impact of resveratrol on inflammation and progression in CRC cells *in vitro* and *in vivo*

Signal modulating networks	Kind of test, CRC cell- /animal Type	Mode of action	References
*Suppression of pro-inflammatory cytokines in CRC*	*In vitro,* HCT-116, HCT-116R cells	Modulation of TNF-β signaling pathway, suppression of NF-κB activation	[80]
	*In vitro and in vivo,* LoVo cells and Mice	Inhibition of inflammation, EMT through TGF-β1/Smads signaling, suppression of metastasis and plasticity in CRC cells	[196]
	*In vitro,* HCT-116/ RKO/SW480 cells	Inhibition of TNF-β/TNF-β-receptor-induced activation of NF-κB, NF-κB-promoted gene products	[199]
	*In vivo,* Rat	Inhibition of neutrophil infiltration, cytokines and oxidative stress, reduction of colitis	[215]
	*In vivo,* Mice	Modified gut microcosm leads to anti-inflammatory response and alleviation of inflammation-promoted CRC	[216]
*Suppression of inflammation, proliferation and tumor formation / induction of apoptosis in CRC*	*In vitro,* HT-29/WiDr cells	Inhibition of inflammation and proliferation in CRC cells	[217]
	*In vitro,* HCT-116 cells	Inhibition of Wnt/survivin signaling, inflammation, activation of p53-independent apoptosis	[218]
	*In vitro,* HCT-116/RKO/SW480 cells	Inhibition of TNF-β/TNF-βR-induced EMT via suppression of NF-κΒ, FAK and plasticity	[196, 207]
	*In vitro,* HCA17/SW480/HT-29 cells	Inhibition of inflammation, proliferation and apoptosis in CRC cells	[219]
	*In vitro,* HCT-116/Caco-2 cells	Inhibition of proliferation by inducing G1/S-phase cell cycle arrest, apoptosis by caspase/cyclin-CDK pathways	[220]
	*In vitro,* HCT-116/CO115 cells	Activation of p53-mediated apoptosis, inhibition of inflammation	[221]
	*In vitro,* DLD1/HCT-15 cells	Inhibition of inflammation and proliferation by targeting the Akt/STAT3 signaling pathway	[123]
	*In vivo,* Rat	Inhibition of DMH-induced irregular crypt lesion foci, CRC formation, increased expression of anti-oxidant enzymes	[222]
	*In vivo,* Mice	Inhibition of CRC formation and inflammation	[89]
	*In vivo,* Rat	Inhibition of CRC occurrence through regulation of inflammatory enzymes, growth of aberrant crypt lesions	[223]
	*In vivo,* Rat	Inhibition of precancerous lesions in the colon and inflammation	[224]
	*In vivo,* Mice	Inhibition of intestinal tumorigenesis by reducing genes involved in cell proliferation and inflammation	[225]
	*In vitro and in vivo,* HCT-116 cells and Mice	Resveratrol with curcumin inhibited CRC cell growth more effectively than either agent alone, associated with reduction of inflammation, proliferation, stimulation of apoptosis, together with a decrease in NF-κB activity	[226]
	*In vivo,* Mice	Inhibition of PGE_2_, COX-2 expression and the number of adenomas	[227]
	*In vivo,* Rat	Inhibition of CRC, inflammation and aberrant crypt foci by targeting Bax and p21 expression	[228]
	*In vivo,* IRC mice	Inhibition of iNOS, NF-κB, STAT3, and ERK expressions	[208]
	*In vitro,* HCT-116 and DLD1 cells	Inhibition of Akt/STAT3 and NFκB	[209]
	*In vitro,* DLD1 and HCT-15 cells	Inhibition of Akt/STAT3, cyclin D1, cyclin E2, and Bcl-2, increase of p53	[123]
	*In vitro,* HT-29 and SW-620 cells	Inhibition of Akt and STAT3, induction of ROS	[210]
	*In vitro,* HCT-116 cells	Inhibition of COX-2 in both miRNA and protein levels	[211]
	*In vitro,* HT-29 cells	Inhibition of NO and PGE_2_ production, iNOS and COX-2 expression and ROS, suppression of JAK/STAT and SAPK/JNK pathways	[212]
	*In vivo,* HT-29 colon cancer xenograft using nude mice	Resveratrol+ginkgetin enhanced the anti-tumor effect of 5-FU via decrease in microvessel density of tumors, suppressing expressions of COX-2, TNF-α and IL-6	[213]
	*In vitro* and *in vivo,* 2D and 3D model of HCT-116 cells murine subcutaneous xenograft HCT-116 model	Micro-immunotherapy medicine + resveratrol induced immunomodulation of macrophages, decrease in the volume of 3D spheroids, decrease in the volume of xenografts	[214]

Abbreviations: *5-FU* 5-fluorouuracil, *Akt* protein kinase B, *Bax* bcl-2-like protein 4, *Bcl-2* B-cell lymphoma 2, *CDK* cyclin-dependent kinase, *COX-2* cyclooxygenase 2, *CRC* colorectal cancer, *DMH* 1,2-dimethylhydrazine, *EMT* epithelial-to-mesenchymal transition, *ERK* extracellular signal-regulated kinases, *FAK* focal adhesion kinase, *iNOS* inducible nitric oxide synthase, *JAK* janus kinase, *NF-κB* nuclear factor kappa-light-chain-enhancer of activated B-cells, *PGE*_*2*_ prostaglandin E_2_, *IL* interleukin, *MMP* matrix metalloproteinase, *NO* nitric oxide, *ROS* reactive oxygen species, *SAPK* stress-activated protein kinase, *STAT3* signal transducer and activator of transcription 3, *TGF* transforming growth factor, *TME* tumor microenvironment, *TNF* tumor necrosis factor

An intense functional collaboration between tumor cells and immune cells is known to exist in the TME, which is essential for the further growth and progressive spread of tumors. Therefore, it is also important that the interaction between both systems is altered in favor of anti-tumor immunity [235]. T-lymphocytes (CD4+), which are among the most important immune cells of the entire defense system, have the ability to differentiate into other T-lymphocytes such as CD8+ and regulatory T-cells (Tregs) in TME in a variable and cytokine-dependent manner, making this interaction a critical factor in slowing tumor growth and providing a good prognosis for patients [236, 237]. Interestingly, resveratrol has been shown to modulate T-cells (CD4+) by excreting interferon (IFN)-γ and thereby up-regulating its biotarget Sirt-1 in CD4+ T-cells [238]. Moreover, these tumor-specific cytotoxic cells (CD8+ T-cells) can attack cancer cells through apoptosis-specific factors (IFN-γ, IL-4, and TNF-α) and mechanisms (perforation of the membrane) and initiate active suicide of tumor cells [239], promoted by resveratrol by participating in anti-tumor immunity, as reported by Choi and colleagues [240].

As previously reported, resveratrol suppresses HDACs, which correlates with the formation of various anti-inflammatory lymphocytes and Treg cells in the gut. In addition, a study in The Cancer Genome Atlas (TCGA) reported that an increase in the activity of the Treg-specific transcription factor FoxP3 or the anti-inflammatory IL-10 contributes to improved survival in patients with CRC. This suggests that changes in the gut microbiome may lead to an anti-inflammatory T-cell response that attenuates inflammation-related CRC [12, 216].

In addition, several investigations showed a significantly enhanced expression and stimulation of NO by iNOS in CRC cells, indicating an essential role of NO in tumorigenesis in the colon cells [241]. Interestingly, resveratrol decreases specific iNOS expression in CRC cells [242]. One of the most essential and primary mechanisms of action of resveratrol is its anti-inflammatory potential, the suppression of the p38-MAPK signaling pathway, which is involved in the production of inflammatory mediators such as cytokines, COX-2, p53, and iNOS [243] and thus is an important requirement for the prevention and inhibition of colitis and CRC (Table 2). For this reason, specific targeting and modulation of pro-inflammatory metabolic pathways, such as NF-κB have significant implications for preventing and eliminating serious diseases [193].

## Resveratrol acts as a chemosensitizer in CRC cells

### Difficulty of chemoresistance in CRC cells

Surgical intervention and chemotherapeutic drug administration remain the main treatment options for CRC patients. However, the decision to use one or more of these approaches in treating CRC subjects depends on the tumor’s location, the cancer stage at diagnosis, and the case-by-case characteristics of the patient [244]. The challenge often arises when a patient (a) shows inherent resistance to the drug due to intrinsic drug resistance capabilities of the cancer cell or (b) develops resistance to a chemotherapeutic drug through exposure to the drug, called acquired resistance [245]. Chemotherapeutic drug resistance leads to lower sensitivity to the drug, compromised drug inefficacy, cancer cell plasticity, tumor relapse, poor prognosis, and higher mortality rates among CRC patients [246–248].

**Fig. 3:**
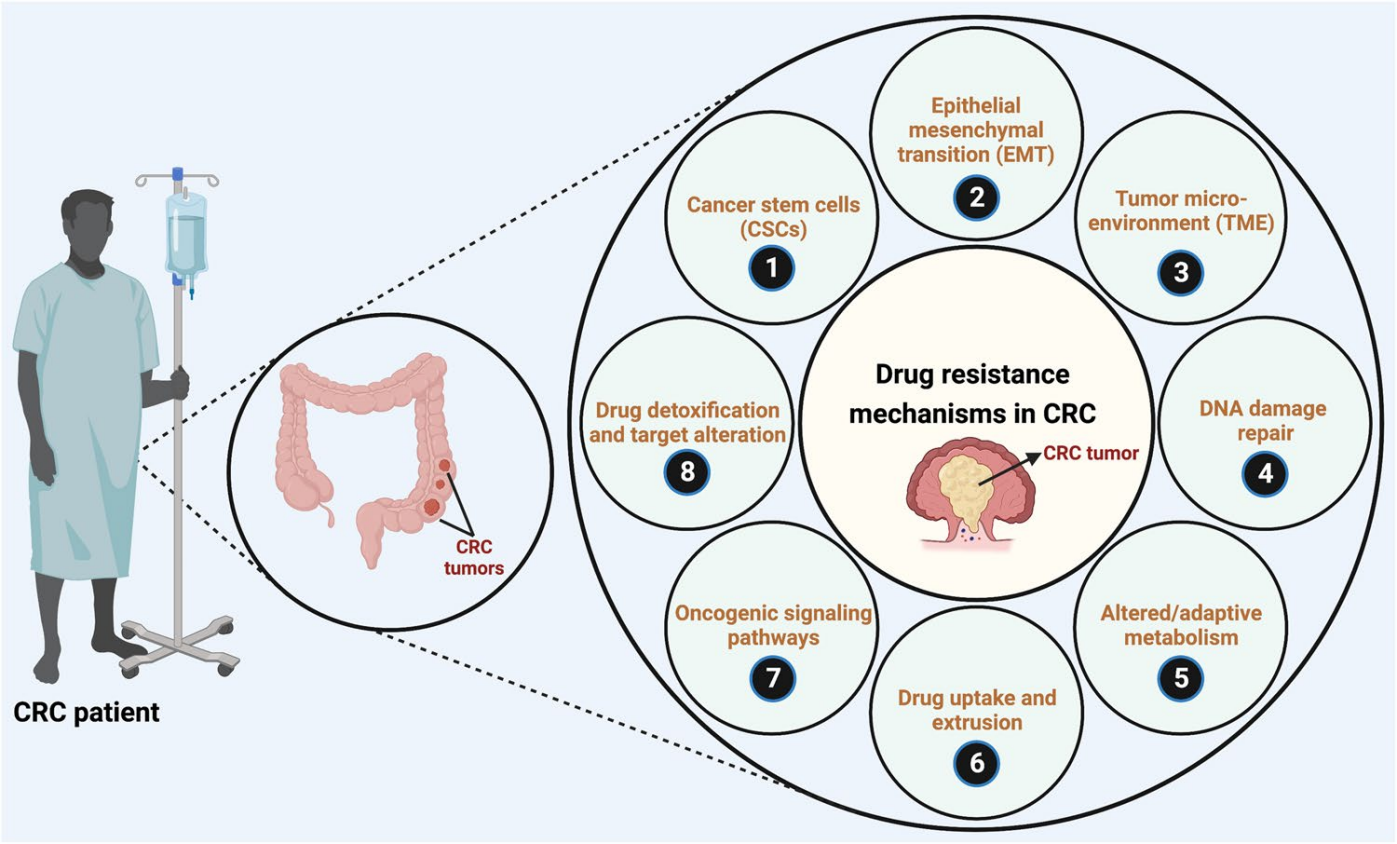
The different mechanisms of chemoresistance in CRC (Figure and Figure legend adapted from Samuel SM, et al., 2020) [245]. The mechanisms of cancer cell plasticity in therapeutic resistance mainly include; (**1**) the presence and influence of cancer stem cells (CSCs) that can initiate and re-populate tumors, (**2**) epithelial–to-mesenchymal transition (EMT), (**3**) tumor microenvironment (characterized by hypoxia, inflammation, autophagy, and presence of cancer-associated fibroblasts, immune cells such as tumor-associated macrophages, and tumor endothelial cells), (**4**) active DNA damage repair mechanisms, (**5**) altered/adaptive/aberrant metabolism (characterized by the Warburg effect, altered amino acid/protein/lipid and nucleotide metabolism, utilization of glutamine, and isoforms of metabolic enzymes that support cancer initiation, progression, and resistance to therapy), (**6**) variations in drug uptake and active drug extrusion systems (ATP binding cassette; ABC/multidrug transporters), (**7**) activation of oncogenic, pro-survival and anti-apoptotic signaling pathways (PI3K, phosphatidylinositol-3-kinase; Akt, protein kinase B; mTOR, mammalian target of rapamycin; MAPK, mitogen activated protein kinase; NF-κB, nuclear factor kappa-light-chain-enhancer of activated B-cells; Wnt/β-catenin; JAK, janus kinase; STAT3, signal transducer and activator of transcription 3; HIF-1, hypoxia inducible factor 1 pathways), and (**8**) active drug detoxification and target alteration systems. Created with BioRender.com

One or more non-cellular (such as limited vascular accessibility and TME) and cellular (such as drug targets, levels and activity of detoxifying enzymes, levels, and activity drug uptake and extrusion transporters) factors play vital roles in CRC drug resistance [244]. Detailed discussions on the various mechanisms of chemotherapeutic drug resistance are beyond the scope of this current review article. Several articles have reported the multiple aspects and mechanisms of chemoresistance in CRC (illustrated in Fig. 3) and novel strategies to reverse resistance extensively and thoroughly [249–251].

The most widely used chemotherapeutic drugs for treating CRC include 5-FU, oxaliplatin, doxorubicin, cetuximab, irinotecan, and various combinations of the same. Drug resistance has been reported against one or more of these drugs and is summarized in Table 3 [252–276]. In some instances, however, CRC resistance to drugs such as oxaliplatin enhanced the sensitivity to tumor necrosis factor-related apoptosis-inducing ligand (TRAIL) via the up-regulation of death receptor 4 and localization of lipid rafts [277]. Interestingly, a significant reduction in viable circulating tumor cells (CTCs) was observed in metastatic CRC patients treated with TRAIL liposomes [277].

**Table 3:** Selected drug resistance in CRC: molecular basis and mechanisms

Drug	Pathway or related enzyme/protein	Type of study	Effects and possible mechanisms of chemoresistance	References
5-Fluorouracil (5-FU)	Thymidylate synthase (TS)	Meta-analysis, 20 studies, 3497 CRC patients	↑TS expression leading to; ↑5-FU target inhibition and ↓5-FU sensitivity	[249, 252]
		*In vitro*, SNU-C1 parental and SNU-C1 (5-FU resistant)	↑TS mRNA expression; ↑TS activity	[253]
		Clinical, formalin-fixed paraffin-embedded specimens, 132 CRC patients (Dukes’ B=36 cases, Dukes’ C=60 cases, Dukes’ B=36 cases)	↑TS expression correlated to 5-FU resistance, shorter recurrence free-interval, and reduced overall survival	[254]
		Clinical, tissue samples (normal, primary, and liver metastases), digital karyotyping	↑Gene amplification of TS major mechanism of 5-FU resistance	[255]
		Clinical, PCR analysis of genomic DNA, 121 CRC patients	↑TS expression associated with poor response to 5-FU	[256]
	Inhibitor of DNA binding 1, HLH protein (ID1)	*In vitro*, CRC-stem-like cells; *In vivo*, mice xenograft model	↑ID1 (stemness marker) expression	[257]
	p53 related apoptosis	*In vitro*, HCT-116	↓p53 correlated to; ↑5-FU resistance and ↓apoptosis	[258]
	RhoGDI_2_ and apoptosis	*In vitro*, LoVo	↑RhoGDI_2_; ↑CapG; ↓Maspin; ↓Apoptosis	[259]
	SHMT2 and autophagy	Clinical, paired-frozen-primary samples (CRC and adjacent normal tissue), 50 patients; clinical, paraffin-embedded samples, 378 patients; *In vivo*, mouse xenograft model	↓SHMT2; ↓SHMT2 binding of cytosolic p53; ↓Apoptosis; ↑Pro-survival autophagy, ↑Plasticity	[196, 260]
	p38-MAPK, apoptosis, and autophagy	*In vitro*, RKO, HT-29, LoVo, SW620 and HCT-116	↓p38-MAPKα; ↓Apoptosis; ↑Autophagy	[261]
	RAC3, apoptosis, and autophagy	*In vitro*, HT-29, LoVo and HCT-116	↑RAC3; ↓Apoptosis; ↓Autophagy	[262]
	TGF-β and EMT	*In vitro*, HCT-116, HCT-116p53KO and HT-29; *In vivo*, mice xenograft model	↑TGF-β; ↑Proliferation; ↓Cell death; ↑Plasticity	[263]
	OPRT-RR and TP-TK pathways	*In vitro*, SW48 and LS174T (parental and resistant)	In SW48-5-FUR cells - ↓OPRT; ↓TP; ↓FdUMP; ↓Drug sensitivity; ↑IC_50_ In LS174T-5-FUR cells - ↓OPRT; ↓RR; ↑TK; ↑dTMP; ↓Drug sensitivity; ↑IC_50_	[264]
	Hedgehog pathway	*In vitro*, LoVo (parental and resistant)	↑GLI1; ↑IC_50_ for 5-FU	[265]
Irinotecan (SN-38; irinotecan metabolite)	ABCC1/MRP1, ABCC2/MRP2, ABCG2/BCRP, multi-drug resistance protein (MDR/MRP)	*In vitro*, HCT-116	↑Expression of MDR/MRP proteins; ↑Drug efflux; ↑Plasticity	[196, 266]
	ABCG2/BCRP, multi-drug resistance protein (MDR/MRP)	*In vitro*, HCT-116, S1-IR20 (novel irinotecan resistant CRC cell line)	↑ABCG2/BCRP; ↑Drug efflux	[267, 268]
	ATP7A, copper transporter	Clinical, 50 patients with advanced CRC	↑ATP7A; ↑Drug efflux; ↑Drug uptake in membrane vesicles	[269]
Oxaliplatin	OCT, drug intake	*In vitro*, LS180, SW620, DLD, HT20, RKO and HCT-116	↓OCT1 (SLC22A1); ↓OCT2 (SLC22A2); ↓Oxaliplatin sensitivity	[270]
	CHK2, DNA repair	*In vitro*, HT-29, LoVo, Colo201 and Colo205; *In vivo*, mice xenograft model	↑phosphorylated CHK2 (pCHK2T68); ↑Homologous recombination repair pathways; ↑DNA repair; ↑CHK2/PARP1 interaction	[271]
	ABGC2, a multi-drug resistance protein (MDR/MRP)	*In vitro*, LoVo (parental and resistant)	↑ABGC2; ↓G2 cell-cycle arrest; ↑phosphorylated NF-κB; ↓ER stress; ↓Apoptosis	[272]
	Nrf2, oxidative stress	*In vitro*, HCT-116 and SW620	↑Nrf2; ↑Nrf2 regulated gene expression; ↓Proliferation; ↑IC_50_ for various drugs	[273]
Cetuximab	KRAS WT tumors	Clinical, 220 chemorefractory metastatic CRC (cmCRC) patients	↑EREG; ↑AREG; ↓Survival	[274]
	EGFR somatic sequence alterations (G465R, G465E, S468R, S492R)	*In vitro*, LIM1215 and OXCO-2	↓mAb binding; ↓EGFR pathway inhibition	[275]
	EGFR mutation (pS492R)	Clinical; plasma samples from 1010 patients with metastatic CRC	↓mAb binding; ↓EGFR pathway inhibition	[276]

The upward arrow (↑) indicates an activation/increase/up-regulation and the downward arrow (↓) indicates a decrease/down-regulation/suppression. Abbreviations: *5-FU* 5-fluorouracil, *5-FUR* 5-FU resistant, *ABCC1/2* ATP binding cassette subfamily C member 1 or 2, also known as MRP1/2, *ABGC2* ATP binding cassette subfamily G member 2, also known as BCRP, *AREG* Amphiregulin, *ATP7A* ATPase copper transporting 7 alpha, *BCRP* Breast cancer resistance protein, also known as ABGC2, *CapG* Capping actin protein, gelsolin like, *CHK2* Checkpoint kinase 2, *EGFR* epidermal growth factor receptor, *EMT* epithelial-tomesenchymal-transition, *EREG* Epiregulin, *FdUMP* Fluoro-deoxyuridine monophosphate, *GLI1* Glioma-associate oncogene family zinc finger 1, *ID1* Inhibitor of DNA binding 1, *KRAS* Kirsten rat sarcoma, *mAb* monoclonal antibody, *MAPK* mitogen activated protein kinase, *MDR/MDRP* multi drug resistance protein, *MRP1/2* multidrug resistance associated protein 1 or 2, also known as ABCC1/2, *NF-κB* nuclear factor kappa-light-chain-enhancer of activated B-cells, *Nrf2* Nuclear factor erythroid 2 related factor 2, *OCT1* Organic cation transporter 1, also known as SLC22A1, *OCT2*, Organic cation transporter 2, also known as SLC22A2, *OPRT*, Orotate phosphoribosyl transferase, *RAC3* Rac family small GTPase 3, *RhoGDI*_*2*_ Rho GDP dissociation inhibitor 2/β, *RR* Ribonucleotide reductase, *SHMT2* Serine hydroxymethyl transferase 2, *TGFβ* Transforming growth factor beta, *TK* thymidine kinase, *TP* Thymidine phosphatase, *TS* Thymidylate synthase, *WT* Wild-type

### Chemoresistance through tumor cell plasticity in CRC cells

Although chemotherapy is the mainstay of cancer treatment, the lack of efficacy of this treatment is a major concern. For many years, researchers believed that the failure of cancer treatments might be due to intrinsic genomic mechanisms, such as the development of mutations in the drug target that prevent its binding [144, 278]. Nevertheless, the primary cause of the ineffectiveness of chemotherapeutic agents is the acquisition of drug resistance by tumor cells throughout treatment [279]. Therefore, it is vital to understand the mechanisms of drug resistance to develop more efficacious treatments that can reduce the risk of relapse. An important factor that is implicated in drug resistance is the plasticity of tumor cells [143]. Cellular plasticity, concerning tumorigenesis, refers to the capacity of terminally differentiated cancer cells to undergo drastic changes in their cell phenotypes in response to oncogenic drivers or external stimuli [145]. The different types of plastic behaviors that help tumor cells acquire drug resistance are EMT, otherwise called epithelial-mesenchymal-plasticity, attaining properties of cancer stem cells (CSCs), and transdifferentiation into other cell types [143, 144]. A deeper insight into the molecular mechanisms underlying CRC drug resistance has revealed a convincing link between these tumor cell plasticity hallmarks and CRC progression [280, 281].

EMT is a developmental process during embryogenesis, tissue remodeling, and wound healing that allows epithelial cells to attain mesenchymal phenotype to enable its motility and invasiveness [282–284]. However, molecular pathways comparable to development have been observed in cancer cells leading to EMT-plasticity [284]. This transition occurs through transcriptional repression of E-cadherin (cell adhesion molecule) by Snail, Slug, zeb 1/2, smad interacting protein 1 (SIP1) or Twist1, and elevated expression of vimentin and N-cadherin, through a complex network of signaling cascade which facilitates collective cell migration and invasion. Cellular growth factors, including epidermal growth factor (EGF), hepatocyte growth factor (HGF), and transforming growth factor beta (TGF-β), are potent stimulators of EMT, which upon binding to their corresponding receptors, initiate signaling pathways including Notch, β-catenin/Wnt and PI3K/Erk signaling [284]. Further research has demonstrated the link between several EMT markers, apoptosis evasion, and enhanced cancer cell survival [285, 286]. This correlation has stimulated interest among researchers to investigate the connection of these EMT markers with resistance to anti-neoplastic treatment modalities. Apart from initiating tumor and inducing metastasis, EMT also confers resistance to cancer treatment interventions, including radiotherapy [285, 287, 288]. For instance, Liu and co-workers demonstrated that vincristine-resistant colon adenocarcinoma cells showed increased expression of Twist1 and thus exhibited elevated migratory and invasive ability. More importantly, this study showed that up-regulation of Twist1 markedly increased chemoresistance to vincristine by up-regulating the ATP-binding cassette transporters, ABCB1 and ABCC1 [288]. Nonetheless, the mechanism of how EMT-plasticity contributes to drug resistance is not yet fully understood. However, it is believed that the cells become more resistant to pro-apoptotic signals and excessive drug efflux by membrane transporters, leading to cell survival despite treatment [289].

An interesting study identified circulating tumor microemboli (CTMs) and three subpopulations of CTCs, namely, E-CTCs, M-CTCs, and E/M-CTCs based on the expression of epithelial cell adhesion molecule (EpCAM), the mesenchymal cell marker vimentin, or both EpCAM and vimentin respectively from blood samples of 126 CRC patients. However, the results showed that M-CTCs and CTMs were highly detected in patients with lymph node metastasis of CRC [290]. Another investigation explored the active involvement of reactive stroma in the modulation of EMT-plasticity in CRC and TNF-α produced by macrophages accelerated the process of TGF-β-induced EMT. This study also demonstrated an interplay between TNF-α and TGF-β signaling in the morphological conversion of organized colon epithelial cells to scattered mesenchymal cells. Besides, TNF-α stimulated Erk activation, which causes increased production of this cytokine by tumor cells. Hence, the role of stroma in the EMT-plasticity of CRC was further elucidated [291].

Moreover, Bates and colleagues revealed a significant role of the integrin αvβ6 (a receptor for fibronectin and tenascin) in CRC progression and metastasis. Their results showed that αvβ6 activated autocrine TGF-β, which causes EMT-plasticity. Clinical analysis of 488 CRC patient samples exhibited a reduction in survival of patients with increased expression of αvβ6, compared to the patients with low or no β6 expression and suggested that β6 expression could be a potential prognostic variable for CRC [292]. Another study reported that caspase-3 gene knockout caused reduced expression of EMT markers such as N-cadherin, Snail, Slug, and zeb1 and elevated E-cadherin expression and chemosensitivity compared to parental CRC cells. Furthermore, the critical role of caspase-3 in cancer cell invasion and metastasis was indicated [293]. Additionally, another examination demonstrated pre-mRNA processing factor (PRP4) as an essential factor in inducing EMT-plasticity and drug resistance in CRC cells by directly binding to p53 and causing its phosphorylation and up-regulating HIF-1α and miR-210, which activates p53 [294].

Focusing chronic oxaliplatin treatment of CRC cells, this therapy resulted in phenotypic changes associated with cellular plasticity, such as loss of polarity, spindle shape, and increase in mobility of these cells along with a decrease in E-cadherin expression and an increase in the expression of Snail and vimentin. This study showed that chronic oxaliplatin resistance in CRC cells leads them to switch to an invasive phenotype and initiates EMT [295]. Long-term exposure of CRC cells to 5-FU enabled these cells to overcome S-phase arrest, evade apoptosis and activate autophagy which is evident by the up-regulation of LC3B, vimentin, Twist1, Slug, and zeb2 mRNA levels and down-regulation of E-cadherin and Claudin-3 [296]. These results suggested that CRC cells respond to chemotherapy-induced cell stress by undergoing EMT-plasticity as an adaptive mechanism, leading to cell survival, plasticity, and evasion of apoptosis.

Another critical determinant of plasticity in CRC cells is CSC. CSCs are a subset of cells found in tumors that can self-renew, differentiate, and produce all cancer cell types. They are responsible for tumor initiation, maintenance, and recurrence [297]. CSCs are isolated and enriched from different tumors by identifying the CRC-specific expression of cell surface markers, including CD44, CD133, CD166, Lgr5, ALDH1, and EpCAM [298]. Further, they initiate aberrant expression of several cellular signaling pathways to maintain their stemness and self-replenishing properties. For instance, Wnt/β-catenin, Notch, TGF-β, and Hedgehog pathways are well implicated in colon cancer CSC development [299–303].

Colon CSCs are the initiators of tumor cell proliferation, invasion, and metastasis to distinct locations. They are attributed to showing high resistance to chemotherapy and are a fundamental reason for tumor relapse or recurrence [304]. For example, many studies have reported that colon CSCs exhibit increased expression of anti-apoptotic proteins and apoptotic inhibitors, as well as ABC transporter proteins that expel drugs out of cancer cells [305, 306]. Moreover, an interesting study revealed that most circulating or migratory CRC cells are Lgr5- and this plastic behavior has an immense capacity for distant metastasis [307]. Additionally known is that the CD133+ CRC cell population resists anti-angiogenesis therapy, and this resistance occurs through an anti-apoptotic pathway including PP2A, p38MAPK, MAPKAPK2, and Hsp27 [308]. In sum, understanding the biology of CSCs is vital to develop novel therapies that can effectively target this hallmark of tumor plasticity.

Transdifferentiation (lineage plasticity), which refers to the ability of tumor cells and CSCs to switch their phenotypic characteristics into a different cell type, represents a different type of cell plasticity. Accumulating evidence has demonstrated that tumor cells vulnerable to therapeutic drugs transdifferentiate into other specialized cell lineages that are not drug targets [144, 309, 310]. Overall, transdifferentiation is a complex, poorly understood process but is believed to be an essential factor in developing drug resistance to cancer. Therefore, research is needed to understand the differentiation mechanism and its impact on drug resistance in cancer.

### Resveratrol’s chemosensitizing effect by modulation of tumor cell plasticity in CRC cells

Data from different studies have shown that resveratrol sensitizes CRC cells toward chemotherapeutic drugs by modulating their plasticity via many signaling pathways and transcription factors. For example, a fascinating study revealed resveratrol’s suppression of CRC cell invasion and migration by inhibiting the TGF-β1/Smads signaling pathway and EMT. Resveratrol elevated the levels of E-cadherin but down-regulated EMT-inducing transcription factors, Snail and vimentin [196].

Even more interesting, a co-treatment with 5-FU and resveratrol in HCT-116 cells significantly lowered the levels of EMT regulatory factors such as Slug and vimentin and the stemness of the treated cells compared to untreated cells [209]. In accordance therewith, Buhrmann et al. reported that resveratrol treatment chemosensitized HCT-116 CRC cells to 5-FU and induced apoptosis while suppressing NF-κB activation, EMT-plasticity (decreased slug and vimentin, increased E-cadherin) and CSC formation (decreased CD133, CD44, and ALDH1) via the modulation of the TNF-β signaling pathway [80]. Resveratrol (5μM) treatment in 5-FU-resistant CRC cell lines HCT-116R and SW480R and their parental forms (HCT-116 and SW480) blocked cell proliferation and synergistically inhibited 5-FU (0.01-1nM) mediated effects on cell invasion [79]. Resveratrol increased cell-cell contact via increased desmosomes, gap- and tight junctions, and increased the expression of E-cadherin cell adhesion protein in both the parental and 5-FU-resistant forms of the HCT-116 CRC cell line [79]. Interestingly, a significant decrease in the vimentin and Slug (plasticity-associated factors) and the down-regulation of the activation and nuclear translocation of NF-κB (abolishing NF-κB driven gene expression of MMP9 and caspase-3) correlated to the ability of resveratrol to attenuate drug resistance in the 5-FU-resistant CRC cells [79]. Beyond that, the role of oxyresveratrol, a natural derivative of resveratrol, in inhibiting EMT-plasticity and metastasis of CRC cells has also been proven [311].

Similarly, many studies focus on targeting aberrant signaling pathways leading to the proliferation and enrichment of colorectal CSCs. It is well established that mutations in the Wnt/β-catenin signaling pathway, which take place in the stem cells of the intestinal crypt, are crucial for the continued proliferation of cancerous cells and stemness activity in colorectal stem cells [312]. For instance, an intriguing study demonstrated that resveratrol potentiated the anti-cancer effects of grape seed extract against colorectal CSCs in a rodent model by suppressing the Wnt/β-catenin pathway. Besides, the compound also induced mitochondrial apoptosis of colon CSCs by up-regulating the Bax/Bcl-2 ratio, p53, and cleaved PARP [313].

Transdifferentiation of CSCs to endothelial cells is an essential event in vascular bed formation for angiogenesis and metastasis of tumor cells [314]. Pouyafar and colleagues reported that resveratrol, in combination with sulindac (a Wnt-3a inhibitor), reduced the clonogenicity potential of colon CSCs and prevented the differentiation of CSCs to endothelial cells. Moreover, resveratrol treatment reduced angiogenesis factor YKL-40 and autophagy-related genes in CRC cells [315].

The pathogenesis of CRC is a complex process that can be distinguished on the basis of three different phenotypes: Chromosomal instability (CIN), Microsatellite instability (MSI), and CpG island methylator (CIMP). Indeed, intracellular biotargets of resveratrol, including a protein with high binding affinity, quinone reductase 2 (QR2), were found to be significantly overexpressed in CRC defined by CIN, particularly in cells harboring a positive KRAS (Kirsten rat sarcoma viral oncogene homolog) mutation, and by the MSI but not the CIMP phenotype. Analysis of data from Oncomine showed very good agreement between mRNA expression of QR2 and specific CRC causes [316]. In addition, several genes involved in the regulation of apoptosis, such as PMAIP1, BID, ZMAT3, CASP3, CASP7, and FAS, have been shown to be novel targets for gene regulatory treatment with resveratrol [317].

Conclusively, it is evident that resveratrol can target different tumor cell plasticity markers, provides a novel approach for treating CRC, and can potentially be a potent therapeutic agent.

### Resveratrol’s further chemosensitizing effects on CRC cells

Several articles have extensively documented the synergistic effects of resveratrol and its ability to sensitize drug-resistant cancers, including CRC, to therapeutic intervention [318]. The chemosensitizing effects of this phytopharmaceutical in CRC are summarized in Table 4 [319–324].

**Table 4:** Effect of resveratrol in a combinatory therapeutic approach

Drug	CRC cell line/cancer model	Chemosensitizing/resensitizing effect of resveratrol in a combinatory therapeutic approach	References
5-Fluorouracil (5-FU)	DLD1 and HCT-116 cells	↓Akt signaling pathway; ↓Cellular proliferation and migration; ↑S-phase cell-cycle arrest; ↑Apoptosis; ↓Slug and vimentin (EMT signaling factors); ↓Stemness; ↓STAT3 binding to hTERT promoter site, ↓Plasticity	[209]
	HT-29 and SW620 cells	↑Mitochondrial oxidative stress; ↓Akt; ↓STAT3	[210]
	HCT-116 and HCT-116R* cells	↑Apoptosis (caspase-3); ↓Vimentin and slug, while ↑E-cadherin (EMT factors); ↓CSC phenotype (CD133, CD44, ALDH1); ↓TNFβ induced activation of NF-κB, MMP9, CXCR4	[80]
	HCT-116, HCT-116R*; SW480 and SW480R* cells	↓Cell proliferation; ↓Cell invasion; ↑Cell-cell contact (↑ desmosomes, gap- and tight-junctions); ↑E-cadherin; ↓Vimentin and slug; ↓NF-κB activation and nuclear translocation; ↓NF-κB driven genes (MMP9, caspase-3)	[79]
	HCT-116 and HCT-116R* cells	↓β1-integrin/HIF1α axis B activation; ↓TME promoted viability; ↓Proliferation; ↓Colony formation; ↓Invasion tendency; ↓EMT; ↓NF-κB; ↓VEGF; ↓HIF1α; ↓Stem cell markers (CD44, CD133, ALDH1); ↑Caspase-3; ↑Apoptosis	[319]
Cetuximab	HCT-116 and CT-26 (mouse cell line) cells	↓Growth; ↑Cx43 expression and phosphorylation; ↑Gap junction function; ↓Akt; ↓NF-κB, ↓Plasticity	[320]
Doxorubicin (Adriamycin)	Caco-2 cells	↓P-gp and MDR1; ↓Drug-efflux/extrusion from cells; ↓ CYP3A4 and GST (drug metabolizing enzymes); ↑Caspases-3, -8 and -9; ↑Apoptosis	[321]
	HT-29 and HCT-116 cells	↓IC_50_ of doxorubicin; ↑Bax; ↑Apoptosis; ↑S-phase cell-cycle arrest; ↓P-gp, ↓Plasticity	[322]
Oxaliplatin	Caco-2 cells	↓Cell proliferation; ↓Growth; ↓Survivin; ↑PARP cleavage; ↑Caspase-3 activity; ↑Apoptosis, ↓Plasticity	[323]
Drug	CRC cell line/cancer model	Anti-chemosensitizing effect of resveratrol in a combinatory therapeutic approach	References
Oxaliplatin	HCT-116 cells	↑Survivin; ↓Apoptosis, ↓Plasticity	[324]

The upward arrow (↑) indicates an activation/increase/up-regulation and the downward arrow (↓) indicates a decrease/down-regulation/suppression. R* indicates resistant cell line. Abbreviations: *Akt* protein kinase B, *ALDH1* aldehyde dehydrogenase 1, *CSC* cancer stem cell, *Cx43* connexin 43, *CXCR4* C-X-C motif chemokine receptor 4, *EMT* epithelial-to-mesenchymal transition, *hTERT* telomerase reverse transcriptase (human), *MMP9* matrix metalloproteinase 9, *NF-κB* nuclear factor kappa-light-chain-enhancer of activated B-cells, *STAT3* signal transducer and activator of transcription 3, *TME* tumor microenvironment, *TNFβ* tumor necrosis factor β

5-FU is one of the most frequently chemotherapeutic drugs in the treatment of CRC, either as a monotherapy or in the drug combinations such as FOLFIRI (Folinic acid + Fluorouracil l + Irinotecan hydrochloride) and FOLFOX (Folinic acid + Fluorouracil + Oxaliplatin) [325]. Due to several mechanisms, drug resistance to 5-FU has been observed in many CRC patients [325]. Resveratrol seems to be able to re-sensitize the 5-FU-resistant CRC cells, rendering them susceptible to 5-FU intervention. Inhibition of the Akt signaling pathway, induction of S-phase cell-cycle arrest, inhibition of cellular proliferation and migration, and activation of programmed cell death occurred when the HCT-116 CRC cells were treated with a combination of 5-FU (10μM) and resveratrol (25μM) [209]. Furthermore, the combination of 5-FU and resveratrol inactivated STAT3 and blocked STAT3 binding to its hTERT promoter site, thereby blocking telomerase activity in HCT-116 cells [209]. Another study, using HT-29 and SW620 CRC cell lines, reported that resveratrol (100μM) exposure synergistically potentiated the 5-FU (10μM) treatment-mediated inhibition of cellular growth via the induction of mitochondrial oxidative stress and created an imbalance in the intracellular anti-oxidant enzymes [210]. The combination (resveratrol and 5-FU) treatment-induced increase in cellular oxidative stress was attributed to the significant inhibition of oncogenic Akt and STAT3 in the treated cells [210].

The epidermal growth factor receptor (EGFR), frequently overexpressed in malignant cells, is a crucial contributor to cancer proliferation, angiogenesis, inhibition of apoptosis, and metastasis [320]. Hence, the EGFR pathway can be targeted to curb tumor growth. Cetuximab, a monoclonal antibody, targets EGFR, hinders endogenous ligand binding to EGFR, and thus suppresses the phosphorylation and activation of EGFR. Subsequently, disruption in the EGFR-related downstream pathways, such as the Ras-Raf-MAPK and PI3K-Akt pathways, have also been reported [320]. However, mutations caused by NRAS, KRAS, BRAF, PI3KCA, and Akt activation in cancers may confer the cancer cells’ therapeutic resistance to cetuximab and render it a less effective anti-cancer agent [320, 326]. Cancer cells develop resistance to cetuximab via the activation of Akt [320]. Wang et al. showed that resveratrol (5μg/ml) treatment in HCT-116 and CT-26 (murine colon adenocarcinoma) cells abolished the resistance to cetuximab and sensitized the cells to cetuximab (10μg/ml) exposure [320]. Resveratrol treatment in cetuximab-exposed cells up-regulated the expression and phosphorylation of connexin 43 with a resultant increase in cell-cell contact via gap junction function. It inhibited the activation of Akt and NF-κB which is related to the increase in cetuximab treatment associated with the suppression of the growth of the cancer cells [320].

Resveratrol (50–100μM) potentiated the oxaliplatin (1μM) mediated inhibition of Caco-2 CRC cell growth [323]. This growth inhibitory effect of the combination of resveratrol and oxaliplatin also increased the apoptotic death of Caco-2 cells [323]. Furthermore, the conditioned media from resveratrol and oxaliplatin-treated Caco-2 cells, when used to grow human-monocyte-derived macrophages, activated the tumoricidal potential of the macrophages while preventing their immunosuppressive characteristics [323].

The efficacy of a chemotherapeutic intervention and/or resistance to the drug quite often depends on levels of expression of drug extrusion membrane transporters (such as P-gp, MDR1, and BCRP) and the levels and activity of intracellular drug-metabolizing enzymes (such as CYP3A4 and GST) [321]. In a doxorubicin-resistant CRC cell line, Caco-2, increasing concentrations of resveratrol (1–500μM) inhibited the drug extrusion capabilities of P-gp and MDR1 proteins in a concentration-dependent manner [321]. The resveratrol (20μM) exposed Caco-2 cells were susceptible to doxorubicin treatment compared to non-resveratrol exposed doxorubicin treated cells [321]. Resveratrol also inhibited the activity of drug-metabolizing enzymes CYP3A4 and GST in these cells and increased the caspases-3, caspases-8, and caspases-9, indicating activation of apoptotic cell death in the treated cells [321]. The mRNA levels of P-gp, MDR1, BCRP, CYP3A4, and GST were significantly reduced upon resveratrol treatment in the drug-resistant Caco-2 cells [321]. In HCT-116 and HT-29 CRC cells, a combination of doxorubicin and resveratrol significantly reduced the IC_50_ value of the doxorubicin in the cells [322]. The expression of the pro-apoptotic Bax gene and apoptosis significantly increased when the doxorubicin-exposed HCT-116 cells were treated with resveratrol while inducing S-phase arrest in these cells [322]. Resveratrol seems to sensitize the HCT-116 cells to the anti-cancer effects of doxorubicin by blocking the P-gp drug efflux mechanism in these cells [322].

It is, however, noteworthy that certain studies reported contradicting data; that resveratrol (30 or 50μM) reverses the inhibitory effect of oxaliplatin (2 or 5μM) on the mRNA expression and protein levels of anti-apoptotic survivin in HCT-116 CRC cells and thereby abolishes the cytotoxic effects of oxaliplatin [324].

## Insights of clinical resveratrol application in CRC patients

Based on the promising preclinical results, resveratrol’s modulatory impact on CRC was clinically tested. Despite the advanced stage of the tumor and the associated vulnerability of CRC patients, resveratrol supplementation was well tolerated in all studies to date (Table 5). Regardless of the dosage form, there was no toxicity or gastrointestinal problems [35, 327, 328].

**Table 5:** Proven anti-CRC effects of resveratrol in clinical trials

# of CRC Patients	Study phase	Resveratrol application	Resveratrol’s effects	Signaling target	Year of publication	Reference
9	1	5g SRT501 (microparticular, in sachets)/day, for 10–21 day	Resveratrol was well-tolerated and was detected in blood plasma as well as hepatic tissue affected by metastases. It induced an up-regulation (39%) of apoptosis in malignant tissue.	Apoptosis, caspase-3	2011	[327]
20	1	0.5g or 1g (caplets)/day, for 8 days	Resveratrol was well-tolerated and reduced (*p*=0.05) CRC cell proliferation.	Proliferation, Ki-67	2010	[35]
12 selected, 8 completed	1	20mg or 80mg (tablets)/day, for 14 days	Resveratrol was well tolerated and inhibited significantly (*p*<0.03) the CRC initiation in normal colonic mucosa.	Initiation, Wnt signaling gene	2009	[328]

Abbreviations: *CRC* colorectal cancer, *Ki-67* kiel-antigen 67, *Wnt* wingless-related integration site

Interestingly, a relatively low dose of the phytopharmaceutical (tablets containing 20mg or 80mg resveratrol) significantly reduced the expression of the Wnt gene in the mucosa of the colon cells [328]. Moreover, as the Wnt pathway represents a key signaling for CRC initiation [328], this finding could be highly relevant to break the cancerous cycle. This approach is supported by another clinical trial where CRC patients received 0.5g or 1g resveratrol as caplets for 8 days [35]. Here, treatment with the natural polyphenol inhibited the detection of cell proliferation parameter Ki-67 [35]. Furthermore, the detection of resveratrol uptake in blood and liver affected by metastases succeeded [327]. In malignant hepatic tissue, the phytopharmaceutical induced apoptosis in metastasized CRC cells [327].

Apart from metastasis, chemoresistance is also a common issue in the advanced stage of the disease, so it is currently being investigated with great interest whether resveratrol increases the susceptibility of CRC patients to classic chemotherapeutic agents.

Data from ClincalTrials.gov (https://clinicaltrials.gov/) showed a list of nineteen (search performed on 28 March 2023; using keywords ‘cancer’ and ‘resveratrol’) resveratrol administration-based clinical trials in different cancers. Out of the nineteen trials, there were only three clinical trials pertaining specifically to colon/colorectal cancer and resveratrol intervention’s therapeutic effect/efficacy in these patients (Table 5). Interestingly none of the studies aimed at studying the ability of resveratrol to overcome chemoresistance. Given the abundance of *in vitro*/*in vivo* data pointing towards the therapeutic efficacy of resveratrol and its ability to re-sensitize cancers to a drug intervention in treating CRC, more clinical trials are warranted to determine whether the therapeutic chemosensitizing and anti-plasticity effects of resveratrol can be translated to clinical use.

## Outlook and future perspectives

In treating CRC, chemotherapy represents one of the most important and best therapeutic options by present-day standards. This kind of therapy is highly specialized, mono-targeted, and expensive. In recent years, conventional drug strategy has achieved relatively low efficacy with many severe side effects [329], mainly due to the development of chemoresistance, which represents a significant obstacle to their use [330].

This resistance of cancer cells is based on different molecular pathways that give them key advantages in resisting the applied chemotherapeutic drugs, such as impairment of mitochondria and associated disruption of the respiratory chain. Also, an impairment of the endoplasmic reticulum (ER) and associated disruption of ER functions, impairment of glucose metabolism and related activation of Warburg action, impairment of lysosomal function, and associated residues of chemotherapeutic drugs [331–334] are of significance.

In this regard, it is known that fundamental causes contributing to the emergence of resistance include poorer drug accessibility, complete signaling alteration in CRC cells, and activation of pathways that promote metastasis formation [207, 329, 330]. Relating to that, a sensitization of tumor cells to conventional drugs seems to be a possible way to overcome the resistance of CRC cells. For this purpose, certain natural plant-based compounds could be administered to optimize the effect of chemotherapeutics by modulating different resistance mechanisms [335, 336].

The secondary plant compound resveratrol enhances the effect of common chemotherapeutic agents such as 5-FU or oxaliplatin in CRC cell treatment by simultaneously influencing numerous cellular signal transduction processes [79, 337]. Unlike conventional drugs, phytopharmaceuticals use a poly-targeting strategy, thus preventing the development of acquired resistance and blocking intrinsic drug resistance mechanisms in some instances. This article, therefore, discusses the use of resveratrol as an anti-plasticity agent based on its ability to target and regulate multiple pro-oncogenic and tumor-suppressing mechanisms (EMT, CSC) and circumvent and overcome the chemoresistance in CRC. The natural polyphenol is proposed as an alternative co-therapeutic agent in combination with standard chemotherapeutic interventions, and its application would be a novel, innovative way.

Considered as a whole, the use of active plant ingredients in oncology has many advantages. They represent a unique, virtually inexhaustible source in nature with their mechanisms of action based on a millennium of experience, development, and adaptation. Their function as chemosensitizers is mainly performed by enhancing the effects of conventional drugs in cancer cells, inhibiting tumor-promoting inflammation, repressing cancer cell plasticity triggering their cell death by activating pro-apoptotic targets and inhibiting anti-apoptotic targets, and causing DNA damage. Non-physiologic inflammation represents a critical initiator and promotor of carcinogenesis and thus can substantially affect the therapy in cancer patients. Resveratrol demonstrates significant anti-inflammatory characteristics, which have the potential to enhance the therapeutic outcome and prevent the resistance of CRC cells to chemotherapy drugs in oncology practice. Indeed, resveratrol is a chemosensitizing agent in CRC cells that reduces inflammation response via decreased levels of phosphorylated NF-κB, JAK/STAT3, MMPs, and COX-2 signaling, and pro-inflammatory cytokine levels [79, 80, 209, 338]. All these effects can enhance the toxic properties of conventional anti-cancer drugs and create a kind of synergy with the standard chemotherapeutics, thus leading to anti-resistance mechanisms [64, 65].

Moreover, good tolerability in patients with the natural-component material should be emphasized in this context [339]. A sometimes-cited criticism of the use of phytopharmaceuticals, including resveratrol, is the low bioavailability in the human body. It is undisputed but known that continuous oral supplementation can achieve the desired health-promoting quantities [340]. Similarly, the realization of a piperine-coupled multiplication of resveratrol’s bioavailability [341] could be enriching.

Overall, a majority of the work that has been done has shown the chemosensitization effects of herbal agents such as resveratrol. Still, preventive and clinical approaches are needed to establish whether the above combinations have synergistic effects during direct application on CRC patients. Based on the great need for complementary therapeutic strategies and the favorable availability of secondary plant polyphenols, this area of research will expand rapidly in the future to provide effective treatments for CRC chemoresistance.

## Conclusion

Based on comprehensive preclinical research, resveratrol exerts chemoprotective and chemosensitizing effects through anti-plasticity, anti-oxidant, anti-inflammatory, and pro-apoptotic modes of action. In addition to its anti-cancer efficacy, resveratrol could work as a mitigating agent in chemotherapy-induced toxicities in normal cells/tissues.

In this article, we have shown that the polyphenol resveratrol could be used not only in the prevention of CRC but also as an anti-cancer agent (targeting multiple mechanisms) in conjunction with conventional chemotherapeutic agents to enhance their therapeutic effects and reduce chemoresistance effects through additive and synergistic effects.

Furthermore, we show here that the molecular targets of chemopreventive resveratrol are similar to those currently used for treating CRC, highlighting the importance of more clinical trials to validate its anti-cancer efficacy and reliability in a clinical setting. Using resveratrol as a cancer chemoprotector or chemosensitizing agent combined with conventional chemotherapeutics in CRC patients needs further in-depth clinical evaluations to elucidate discrepancies between the preclinical cancer research and clinical practice. These studies must (a) include precise analysis of pharmacokinetic parameters of resveratrol in humans; (b) find sufficient and safe dosing of resveratrol when used as a monotherapy and/or in combination with existing chemotherapeutics; (c) find the effective combinations of resveratrol with conventionally used chemotherapeutic drugs to re-sensitize the chemo-/radiotherapy-resistant cancers; (d) provide a detailed description of the cellular targets of resveratrol and its effect on specific problems of oncology such as disease relapse and therapy resistance; (e) provide an understanding on how specific individual characteristics of a cancer patient (which can vary from one patient to another) may affect the therapeutic efficacy, chemosensitizing potential and prognosis of resveratrol formulations among treated CRC patients; and (f) asses advanced drug formulations and improved drug deliver techniques, such as nanotechnology, to specifically target neoplastic cells/tissue and this avoid any possible off-target and side-effects of resveratrol when used as a monotherapy and/or in combination with existing chemotherapeutics.

Overall, this review summarizes, for the first time to our knowledge, the chemosensitization of tumor cells to chemotherapeutic agents by resveratrol from the perspective of cellular plasticity, which is of enormous importance for cellular adaptations to the TME during cancer cell transformation and metastasis. It also provides a new impetus for further research into cancer epigenetics and resveratrol-mediated suppression of plasticity, which is a strong indication that resveratrol, an active phytochemical, will play an influential role in the prevention and treatment of CRC in the future.

## Data Availability

All data are available in the manuscript.

## Abbreviations

5-LOX: 5-lipoxygenase
5-FU: 5-fluorouracil
AP-1: activator protein 1
Bcl-2: B-cell lymphoma 2
Bcl-xL: B-cell lymphoma extra-large
CIMP: CpG island methylator
CIN: chromosomal instability
COX: cyclooxygenase
CRC: colorectal cancer
CSC: cancer stem cell
CTC: circulating tumor cell
CTM: circulating tumor microemboli
DNA: deoxyribonucleic acid
EGF: epidermal growth factor
EGFR: epidermal growth factor receptor
EMT: epithelial-to-mesenchymal transition
EpCAM: epithelial cell adhesion molecule
ER: endoplasmic reticulum
HDAC: histone deacetylase
HGF: hepatocyte growth factor
HIF: hypoxia-inducible factor
IkBα: NF-κB inhibitor alpha
IBD: inflammatory bowel disease
IFN: interferon
IL: interleukin
iNOS: isoenzymes of NO synthase
Ki-67: Kiel-antigen 67
MAPK: mitogen-activated protein kinase
MDR: multidrug resistance
MMP: matrix metalloproteinase
mRNA: messenger ribonucleic acid
MRP: MDR-associated protein
MSI: microsatellite instability
NF-κB: nuclear factor kappa-light-chain-enhancer of activated B-cells
NO: nitric oxide
Nrf2: nuclear factor erythroid 2–related factor 2
p53: cellular tumor antigen p53
PGE_2_: prostaglandin
P-gp: p-glycoproteins
PRP4: pre-mRNA processing factor
QR2: quine reductase 2
ROS: reactive oxygen species
SIP1: smad interacting protein 1
SIRT: sirtuins
STAT: signal transducer and activator of transcription
TGF-β: transforming growth factor beta
Th17: *T helper 17* cells
TME: tumor microenvironment
TNF: tumor necrosis factor
TPA: 12-o-tetradecanoylphorbol-13-acetate
TRAIL: tumor necrosis factor-related apoptosis-inducing ligand
Tregs: regulatory T-cells
VEGF: vascular endothelial growth factor.
